# Precise Focal Spot Positioning on an Opaque Substrate Based on the Diffraction Phenomenon in Laser Microfabrication

**DOI:** 10.3390/mi14122256

**Published:** 2023-12-18

**Authors:** Xian Jing, Pengju Zhao, Fuzeng Wang, Mingkun Han, Jieqiong Lin

**Affiliations:** 1 College of Electronic Science and Engineering, Jilin University, Changchun 130012, China; 2 Jilin Provincial Key Laboratory of Micro/Nano and Ultra-Precision Manufacturing, School of Mechatronic Engineering, Changchun University of Technology, Changchun 130012, China

**Keywords:** two-photon lithography, focal spot positioning, laser microfabrication, diffraction patterns, opaque substrate

## Abstract

The precise positioning of the laser focal spot on the substrate is an important issue for laser microfabrication. In this work, a diffraction pattern-based focal spot positioning method (DFSPM) is proposed to achieve the precise positioning of the laser focal spot on opaque substrates. A series of diffraction patterns of laser focus under-positioning, exact positioning and over-positioning were obtained to investigate the cross-section light distribution of the laser focal spot. According to the monotonic tendency of FWHM to exhibit light intensity at the focal spot cross-section away from the focal plane, the FWHM threshold of polynomial fitted curves was used to determine the exact positioning of laser focus. The ascending scanning method was used to obtain the diffraction patterns at various vertical positions and the FWHM threshold of light distribution at the exact position. The polynomial fitted curves verify the FWHM monotonic tendency of light intensity distribution at the focal spot cross-section along the optical axis. Precise positioning can be achieved with a 100 nm adjustment resolution. This work was expected to provide references for laser microfabrication on opaque materials.

## 1. Introduction

The femtosecond laser two-photon lithography is well-known for its nanoscale fabrication resolution beyond the optical diffraction limit and flexible 3D manufacturing capability [1,2,3,4,5]. It has greatly revolutionized the fields of micro/nanofabrication, allowing the production of complex 3D microstructures with flexibility in design, materials, and properties [6,7,8,9,10,11]. A variety of functional devices with delicate structures has been fabricated by the TPL technology and applied in many significant advanced technology fields, such as micro-optics, micro-fluidics, microrobots, microbiological scaffolds, metamaterials and so on [12,13,14,15,16,17,18]. In the TPL, the range of the focal region determines the shape and dimension of fabricated voxels to a large extent [19,20]. The laser beam was tightly focused on the focal region due to the application of a high *NA* objective in the TPL system. The extremely small range of the focal spot leads to a high fabricating resolution but also brings great challenges for the focal spot to be precisely positioned on the substrate. The positioning errors of the laser focal spot on the substrate cause part or all of the fabricated structure to be buried by or separated from the substrate [21,22].

Various methods have been proposed to improve the precise positioning of the focal spot on the substrate in TPL, which can be classified as two approaches according to the referenced medium from fabricating the process or not. The first approach is to locate the laser focal spot position in relation to the substrate plane by processing intermediate materials in addition to the expected structure. This approach can be called the external medium positioning method (EMPM). For example, M. T. B. Najam et al. used ink burn-out lines to solve the interference problem caused by the halo near the focal imaging so as to precisely position the TPL focal spot on the cover slip [23]. Z. Xu et al. used the buried height of the fabricated helical structure to achieve the exact space coordinate of the plane for error compensation [24]. The EMPM has strong versatility and distinct feedback signals, but it also introduces impurities or polymerization structures on the substrate in addition to the expected structure. Correspondingly, the self-medium positioning method (SMPM) utilizes the phenomenon that appears during the fabricating process where, usually, two-photon-induced fluorescence(TPIF) acts as the feedback media to position the focal spot. B. J. Jung et al. used the fluorescence region within the polymerization threshold of the laser focal spot as a form of feedback to achieve autofocus [25]. B. G. Jeon et al. improved the precision and robustness of autofocus by accurately calculating the size of normalized images according to the second momentum radius of TPIF [26]. The premise of all these aforementioned methods is that they can achieve the purpose of observing the TPL process in real-time. However, the existing TPL observation systems mainly adopt transmission illumination. Thus, the TPL process is a black box when the substrate is opaque. This is not conducive to positioning the focal spot in the TPL process.

In this report, a diffraction pattern-based focal spot positioning method (DFSPM) is proposed to achieve precise positioning on a silicon wafer substrate. The photon intensity of the focal spot focusing on various silicon wafer surfaces with the *z*-position was investigated. According to the monotonic tendency of FWHM to produce light intensity at the focal spot cross-section away from the focal plane, the FWHM threshold of polynomial fitted curves was used to determine the exact positioning of laser focus. The ascending scanning method was utilized to obtain the polymerized center of the voxel and the diffraction pattern of the focal spot at the exact positioning location. A series of diffraction patterns at various vertical positions were also obtained to verify the monotonic tendency of the light distribution FWHM. The FWHM difference threshold of polynomial fitted curves was used to determine the exact positioning of laser focus. This work is expected to provide references for the laser microfabrication of opaque materials.

## 2. Materials and Methods

**Figure 1:**
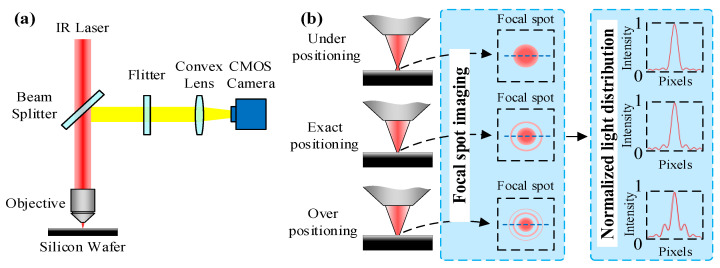
Schematic diagram of DFSPM. (**a**) DFSPM setups. (**b**) Focal spot variation.

The TPL system with the proposed focal spot positioning components is illustrated in Figure 1. An 800 nm infrared Gauss beam irradiated from a femtosecond laser with a pulse width of 100 fs and repetition frequency of 80 MHz was used as the light source. A silicon wafer was used as the substrate. A beam splitter was used to allow the laser beam to pass through and reflect the real-time image on the substrate to the CMOS camera. A short-wave-pass filter was used to reduce the imaging noise. A 100× oil-immersion objective lens with an *NA* of 1.3 was used to focus the laser beam. The *xyz*-axis positioning of the focal spot related to the substrate was achieved via the air-bearing linear stage.

Ormocer (organic modified ceramics) was used as the photoresist in this work. It was sprayed on the substrate. Commercially available OrmoDev^®^ was applied to develop the target structures.

The diffraction phenomenon, which is desperately avoided but still unavoidable in laser extreme manufacturing, was used as a reference for focal spot positioning in this work. The proposed DFSPM relies primarily on the Fraunhofer diffraction pattern on the substrate, which can be achieved at close range with a convergence lens [27]. The diffraction phenomenon makes the infrared laser beam form a series of visual diffraction rings in the focal spot region, as shown in Figure 1. The light intensity distributions of the focal central cross-section are also shown in Figure 1. When the *z*-axis air-bearing linear stage drives the objective lens to move from a far distance towards the substrate plane, the diffraction pattern observed from the CMOS camera changes with the relative positive objective lens to the substrate. The laser focal spot progresses from under-positioning to exact positioning and then to over-positioning. Thus, the position of the laser focal spot relative to the substrate plane can be measured using the variation in the detected image.

## 3. Theory

The electric field of the focal spot can be represented using cylindrical symmetry coordinates, which consist of the transverse direction *r* and propagation direction *z* [28]. The photon intensity distribution *I* of the near-focus spot can be approximated by the following Gaussian model:

(1) $$I\left(r,z\right)=I_{0}\left(\frac{w_{0}^{2}}{w{\left(z\right)}^{2}}\right)e^{-2{\left[r/w\left(z\right)\right]}^{2}},$$

where *I*_0_ is the photon flux intensity at the focal spot center (*r* = 0, *z* = 0) and *I*(*r*,*z*) is the photon flux intensity at the polar coordinate (*r*, *z*). In this description, *w*_0_ and *w*(*z*) represent the size of the beam waist at *z* = 0 and the beam radius in the plane with a distance of *z*.

From Equation (1), the distribution of light intensity *I* at the *z* cross-section away from the focal plane along the *z*-positive direction or negative direction tends to become flat. Additionally, the diffraction patterns of the laser focal spot remain constant when both the laser parameters and the setup are kept constant. Thus, the full width at the half maxima (FWHM) *w_hm_* of light intensity distribution at the focal center can be used to quantify the variation in intensity distribution. *w*_hm_(*n*) is the FWHM of light intensity distribution after the *n*th movement towards the substrate. *W*_th_ is the FWHM threshold value of the light intensity distribution, which can be detected from the monitor when the *z*-axis is located at the polymerized center position. Microrods fabricated via the ascending scanning method can be used to obtain the real *z*-position of the focal spot at the polymerized center. When the *z*-axis moves from the position with the minimum *w*_hm_ to bring the objective and sample closer, the *z*-coordinate of the exact positioning location can be calculated using Equation (2).

(2) $$z_{ex}=z_{0}+\left(n-1\right)d_{\min},\;\mathrm{if}\;w_{hm}\left(n\right)>w_{th}$$

where *z*_0_ is the starting *z*-coordinate, and *d*_min_ is the unit feed rate of every displacement.

## 4. Results and Discussion

**Figure 2:**
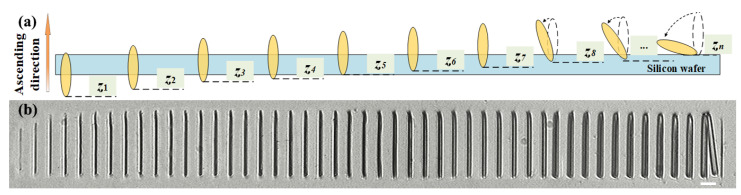
The microrods fabricated by the ascending scanning method. (**a**) Schematic diagram of the ascending scan method at a cross-sectional view. (**b**) Images of microrods fabricated with the rise in the focal spot at *z*-direction. The scale bar is 10 μm.

In our experiment, a series of microrods were fabricated on the silicon wafer via two-photon lithography at different *z*-positions to obtain the real *z*-position of the focal spot at the polymerized center. The air-bearing linear stage drove the objective lens to move towards the silicon wafer substrate at a feet rate of 100 nm along the *z*-axis. The feet rate is one of the key parameters that determines the positioning precision. To improve positioning accuracy, one approach is to subdivide the positioning system in addition to setting a more precise FWHM threshold value. The laser power *P* in front of the objective lens is set as 52 mW. The diffraction patterns of the focal spots were detected using the CMOS camera. The control interface for the *z*-axis and monitoring interface are shown on the same screen and recorded for easy analysis of the corresponding diffraction patterns at the relevant coordinates. A series of microrods were fabricated using the ascending scanning method with a scanning velocity of 20 μm/s. Each microrod was labeled with a fabricated square structure whose length varied according to the assigned number. Figure 2a illustrates the schematic diagram of voxels that were either buried by or separated from the substrate. Figure 2b shows the 48 fabricated microrods that survived after development. It also indicates that the microrod had a vertical length of ~4.8 μm. The different morphologies of the microrods, ranging from just outcropping to almost detached, represent the positioning of the focal spot at different *z*-positions. In the image, the 24th and 25th microrods in the middle represent the center of the polymerized structure. The *z*-positions of the focal spot used to fabricate these microrods indicate the precise location where the laser was focused on the substrate during the experiment. The reason for generating two results is that the most accurate location is between these two locations. The threshold of FWHM *w*_th_ can be determined by taking the average of the FWHM values obtained from the two diffraction patterns at these two positions. In this work, images with 100 pixels × 100 pixels near the center of the focal spot were snipped out to obtain the focal spot data. Polynomials of the 15th order were utilized using the least square method to fit the experimental data and obtain the comprehensive light intensity curve. Polynomial fitting was preferred due to its ability to yield a smaller error when fitting the detected data while requiring less data to accomplish this task. However, it should be noted that the accurate fitting of symmetric functions necessitates more precise spot image data along with additional constraints. After the calculation, *w*_th_ was set as 0.398 μm.

**Figure 3:**
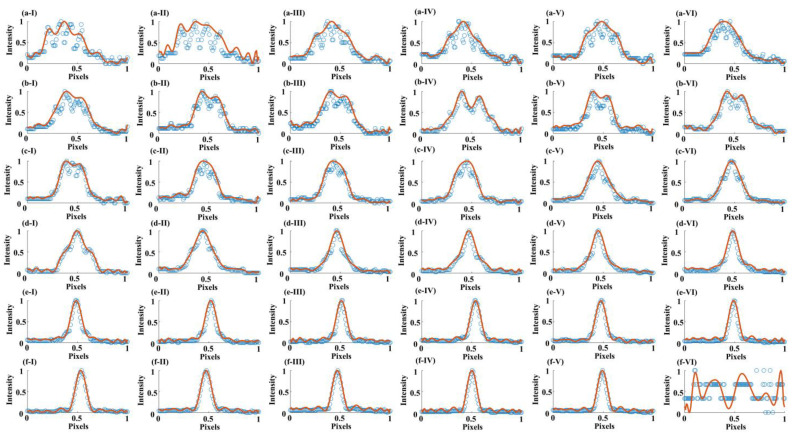
Normalized intensity curves of the focal spot central cross-section at various *z*-positions. The blue hollow dots represent the detected light intensity data of the focal spot while the red curves represent the fitting curves. (**a-I**–**f-VI**) The intensity curves of focal spot when the focus position gradually ascending along the z-axis with a 100 nm interval.

**Figure 4:**
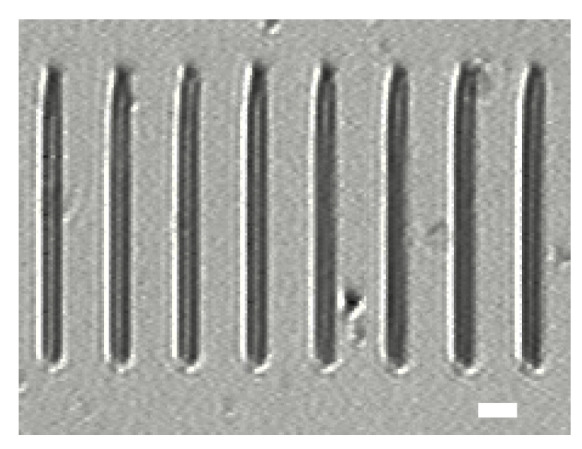
Microrods fabricated with the chosen diffraction pattern. The scale bar is 2 μm.

The light intensities of the central cross-section of the laser focal spot of another group are normalized and shown in Figure 3 with blue hollow dots. Figure 3 shows the normalized intensity curves (in red) of the focal spot central cross-section at 36 *z*-positions. The FWHM of the center of the focal spot’s reach at the substrate surface is shown in Figure 3(d-VI), whose *w*_hm_ = 0.41 μm. Figure 4 displays the fabricated microrods that were created using the chosen diffraction pattern. All the microrods are precisely positioned on the surface of the silicon wafer.

## 5. Conclusions

In this report, a DFSPM was proposed to achieve the precise positioning of the laser focal spot on the opaque substrate based on the diffraction patterns. The effects of under-positioning, exact positioning, and over-positioning on the light intensity distribution of diffraction patterns were investigated. A series of diffraction patterns were obtained to investigate the cross-section light distribution of the laser focus. According to the monotonic tendency of FWHM, the FWHM threshold was used to determine the exact positioning of laser focus. The positioning accuracy was verified by a series of adequately positioned microrods on the silicon wafer. The precise positioning can be achieved with a 100 nm adjustment resolution. This work is expected to provide references for the fabrication of opaque materials and improve the dimensional controllability of the minimum fabricating unit.

## Data Availability

The data that support the findings of this study are available from the corresponding author upon reasonable request.
